# Atypical psychosis in a patient with intracranial hypertension: clinical case and review

**DOI:** 10.1192/j.eurpsy.2024.1015

**Published:** 2024-08-27

**Authors:** B. Lázaro Alonso

**Affiliations:** Psychiatry and Mental Health, Hospital Universitario Ramón y Cajal, Madrid, Spain

## Abstract

**Introduction:**

Several neurologic conditions can produce or mimic psychotic symptoms. It is important to make an exhaustive differential diagnosis between a psychiatric manifestation of an underlying neurological condition and a primary psychiatric one. We explore through the present clinical case of a young woman admitted to neurology the relationship between intracranial hypertension and a case of atypical psychosis that resolved itself with the treatment of the intracranial hypertension, without the need for antipsychotic medication.

**Objectives:**

To explore through the presented clinical case and the concerning literature the concept and management of psychotic-like symptoms in patients with intracranial hypertension.

**Methods:**

We present a clinical case and a review of the existing literature concerning atypical psychosis or psychosis-like symptoms in cases of intracranial hypertension.

**Results:**

We report the case of a 24 year old woman with no relevant medical history hospitalised in the neurology unit due to suspected encephalitis. Native to New Zealand, she is brought from the airport due to behavioural alterations. During the last few days before admission she had presented with incoherent speech, derailment, religious and persecutory delusions, and erotomania towards her cousin. She described feelings of strangeness with her surroundings and of time moving at a different speed than usual, either faster or slower. She also had a headache and visual alterations, as well as memory errors concerning recent events, clouding of consciousness and inattention.

No fever or other relevant physical symptoms. A lumbar puncture is done, which shows an opening pressure of 37mmHg but no other anomalies. Body CT scan shows no relevant findings. Empirical treatment with dexamethasone is initiated for suspected encephalitis, progressively reducing the dosage until suspension in the following days. During her stay at the hospital she is assessed by ophthalmology, which finds no abnormalities in the eye fundus examination, and psychiatry. A second evacuating lumbar punture is done to reduce intracraneal hypertension. No antipsychotic treatment is initiated: the symptomatology remitted with the lowering of intracranial pressure. At time of discharge, the patient remained asymptomatic without treatment and was able to return home to continue outpatient neurologic study of the etiology of the intracranial hypertension.

Finally, we conduct a review ot the existing literature concerning psychotic and psychosis-like symptoms in patients with intracranial hypertension, to explore the diagnostic and management options of this rare finding.

**Conclusions:**

Our findings point to the existing relationship between intracranial hypertension and psychosis-like symptoms. Further studies on pathogenic mechanisms and therapeutic management are required.

**Disclosure of Interest:**

None Declared

